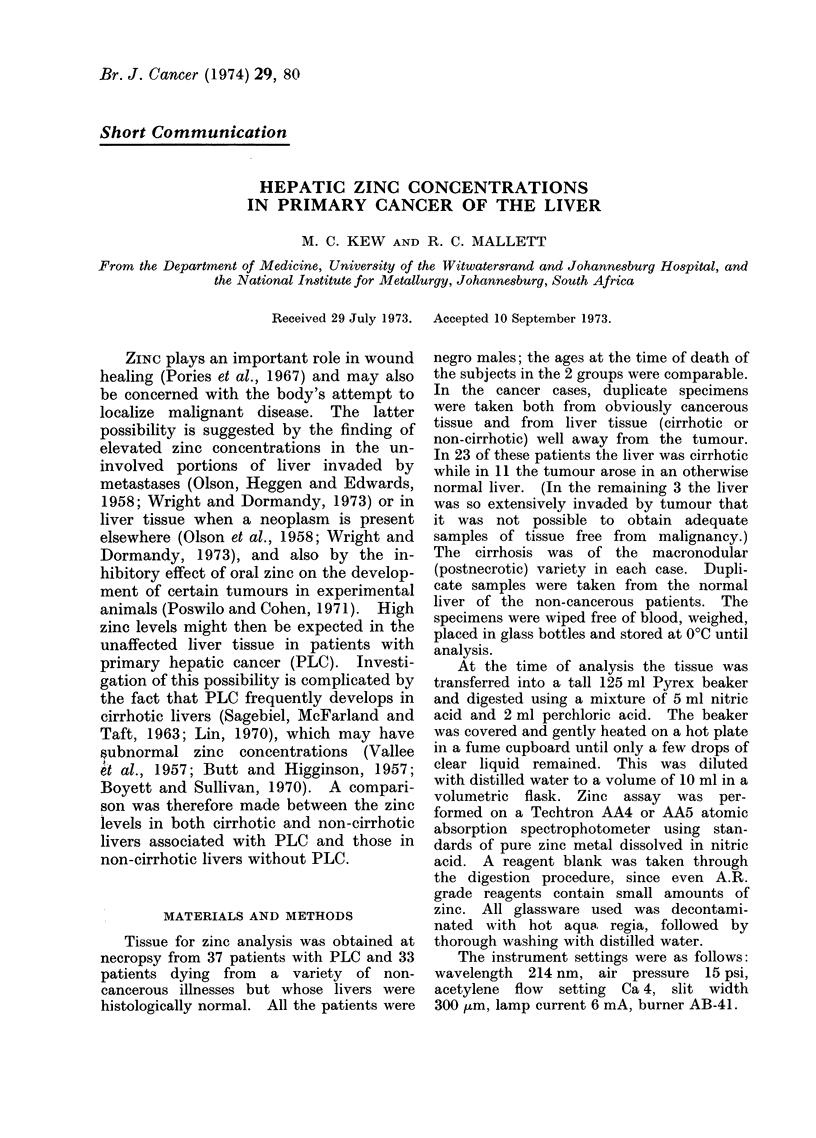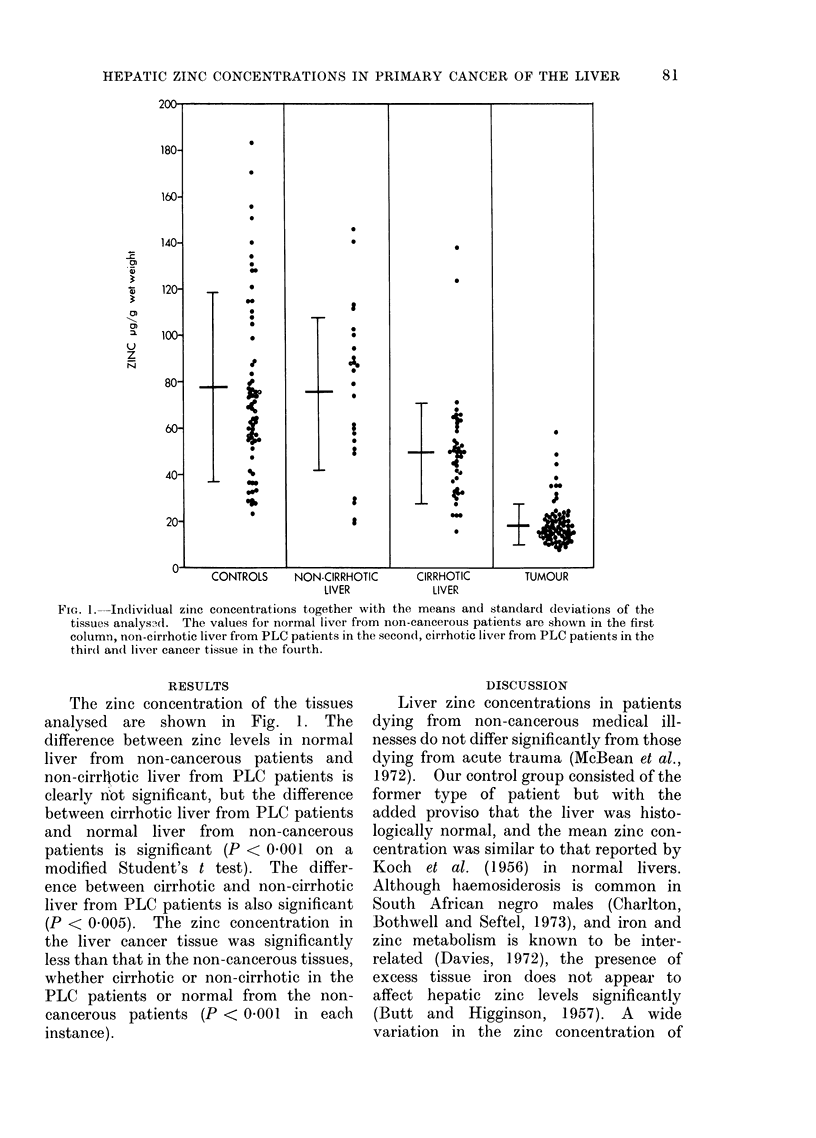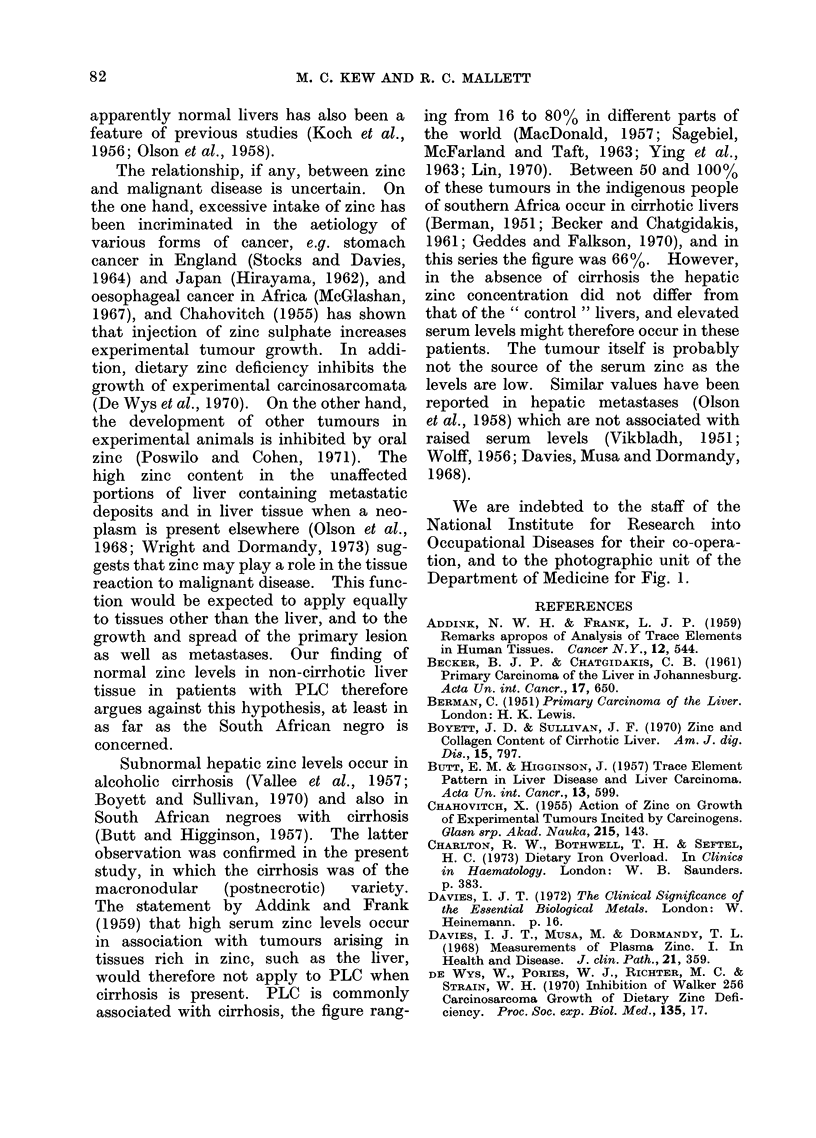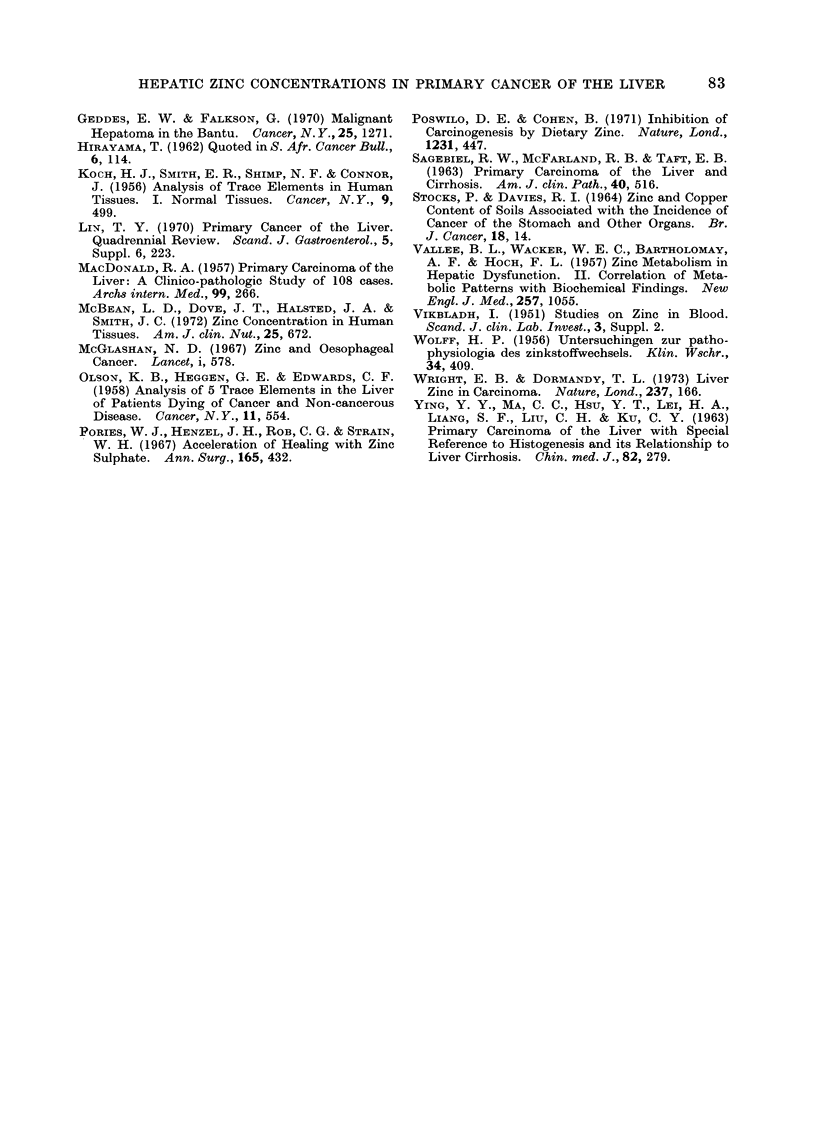# Hepatic zinc concentrations in primary cancer of the liver.

**DOI:** 10.1038/bjc.1974.11

**Published:** 1974-01

**Authors:** M. C. Kew, R. C. Mallett


*Br. J. Cancer* (1974) **29**, 80

***Short Communication***

### HEPATIC ZINC CONCENTRATIONS IN PRIMARY CANCER OF THE LIVER

M. C. KEW AND R. C. MALLETT

*From the Department of Medicine, University of the Witwatersrand and Johannesburg Hospital, and the National Institute for Metallurgy, Johannesburg, South Africa*

Received 29 July 1973. Accepted 10 September 1973.

ZINC plays an important role in wound healing (Pories *et al.*, 1967) and may also be concerned with the body's attempt to localize malignant disease. The latter possibility is suggested by the finding of elevated zinc concentrations in the uninvolved portions of liver invaded by metastases (Olson, Heggen and Edwards, 1958; Wright and Dormandy, 1973) or in liver tissue when a neoplasm is present elsewhere (Olson *et al.*, 1958; Wright and Dormandy, 1973), and also by the inhibitory effect of oral zinc on the development of certain tumours in experimental animals (Poswilo and Cohen, 1971). High zinc levels might then be expected in the unaffected liver tissue in patients with primary hepatic cancer (PLC). Investigation of this possibility is complicated by the fact that PLC frequently develops in cirrhotic livers (Sagebiel, McFarland and Taft, 1963; Lin, 1970), which may have subnormal zinc concentrations (Vallee *et al.*, 1957; Butt and Higginson, 1957; Boyett and Sullivan, 1970). A comparison was therefore made between the zinc levels in both cirrhotic and non-cirrhotic livers associated with PLC and those in non-cirrhotic livers without PLC.

### MATERIALS AND METHODS

Tissue for zinc analysis was obtained at necropsy from 37 patients with PLC and 33 patients dying from a variety of non-cancerous illnesses but whose livers were histologically normal. All the patients were negro males; the ages at the time of death of the subjects in the 2 groups were comparable. In the cancer cases, duplicate specimens were taken both from obviously cancerous tissue and from liver tissue (cirrhotic or non-cirrhotic) well away from the tumour. In 23 of these patients the liver was cirrhotic while in 11 the tumour arose in an otherwise normal liver. (In the remaining 3 the liver was so extensively invaded by tumour that it was not possible to obtain adequate samples of tissue free from malignancy.) The cirrhosis was of the macronodular (postnecrotic) variety in each case. Duplicate samples were taken from the normal liver of the non-cancerous patients. The specimens were wiped free of blood, weighed, placed in glass bottles and stored at 0°C until analysis.

At the time of analysis the tissue was transferred into a tall 125 ml Pyrex beaker and digested using a mixture of 5 ml nitric acid and 2 ml perchloric acid. The beaker was covered and gently heated on a hot plate in a fume cupboard until only a few drops of clear liquid remained. This was diluted with distilled water to a volume of 10 ml in a volumetric flask. Zinc assay was performed on a Techtron AA4 or AA5 atomic absorption spectrophotometer using standards of pure zinc metal dissolved in nitric acid. A reagent blank was taken through the digestion procedure, since even A.R. grade reagents contain small amounts of zinc. All glassware used was decontaminated with hot aqua regia, followed by thorough washing with distilled water.

The instrument settings were as follows: wavelength 214 nm, air pressure 15 psi, acetylene flow setting Ca 4, slit width 300 $\mu$m, lamp current 6 mA, burner AB-41.

FIG. 1.—Individual zinc concentrations together with the means and standard deviations of the tissues analysed. The values for normal liver from non-cancerous patients are shown in the first column, non-cirrhotic liver from PLC patients in the second, cirrhotic liver from PLC patients in the third and liver cancer tissue in the fourth.

### RESULTS

The zinc concentration of the tissues analysed are shown in Fig. 1. The difference between zinc levels in normal liver from non-cancerous patients and non-cirrhotic liver from PLC patients is clearly not significant, but the difference between cirrhotic liver from PLC patients and normal liver from non-cancerous patients is significant ($P < 0{\cdot}001$ on a modified Student's $t$ test). The difference between cirrhotic and non-cirrhotic liver from PLC patients is also significant ($P < 0{\cdot}005$). The zinc concentration in the liver cancer tissue was significantly less than that in the non-cancerous tissues, whether cirrhotic or non-cirrhotic in the PLC patients or normal from the non-cancerous patients ($P < 0{\cdot}001$ in each instance).

### DISCUSSION

Liver zinc concentrations in patients dying from non-cancerous medical illnesses do not differ significantly from those dying from acute trauma (McBean *et al.*, 1972). Our control group consisted of the former type of patient but with the added proviso that the liver was histologically normal, and the mean zinc concentration was similar to that reported by Koch *et al.* (1956) in normal livers. Although haemosiderosis is common in South African negro males (Charlton, Bothwell and Seftel, 1973), and iron and zinc metabolism is known to be interrelated (Davies, 1972), the presence of excess tissue iron does not appear to affect hepatic zinc levels significantly (Butt and Higginson, 1957). A wide variation in the zinc concentration of apparently normal livers has also been a feature of previous studies (Koch *et al.*, 1956; Olson *et al.*, 1958).

The relationship, if any, between zinc and malignant disease is uncertain. On the one hand, excessive intake of zinc has been incriminated in the aetiology of various forms of cancer, *e.g.* stomach cancer in England (Stocks and Davies, 1964) and Japan (Hirayama, 1962), and oesophageal cancer in Africa (McGlashan, 1967), and Chahovitch (1955) has shown that injection of zinc sulphate increases experimental tumour growth. In addition, dietary zinc deficiency inhibits the growth of experimental carcinosarcomata (De Wys *et al.*, 1970). On the other hand, the development of other tumours in experimental animals is inhibited by oral zinc (Poswilo and Cohen, 1971). The high zinc content in the unaffected portions of liver containing metastatic deposits and in liver tissue when a neoplasm is present elsewhere (Olson *et al.*, 1968; Wright and Dormandy, 1973) suggests that zinc may play a role in the tissue reaction to malignant disease. This function would be expected to apply equally to tissues other than the liver, and to the growth and spread of the primary lesion as well as metastases. Our finding of normal zinc levels in non-cirrhotic liver tissue in patients with PLC therefore argues against this hypothesis, at least in as far as the South African negro is concerned.

Subnormal hepatic zinc levels occur in alcoholic cirrhosis (Vallee *et al.*, 1957; Boyett and Sullivan, 1970) and also in South African negroes with cirrhosis (Butt and Higginson, 1957). The latter observation was confirmed in the present study, in which the cirrhosis was of the macronodular (postnecrotic) variety. The statement by Addink and Frank (1959) that high serum zinc levels occur in association with tumours arising in tissues rich in zinc, such as the liver, would therefore not apply to PLC when cirrhosis is present. PLC is commonly associated with cirrhosis, the figure ranging from 16 to 80% in different parts of the world (MacDonald, 1957; Sagebiel, McFarland and Taft, 1963; Ying *et al.*, 1963; Lin, 1970). Between 50 and 100% of these tumours in the indigenous people of southern Africa occur in cirrhotic livers (Berman, 1951; Becker and Chatgidakis, 1961; Geddes and Falkson, 1970), and in this series the figure was 66%. However, in the absence of cirrhosis the hepatic zinc concentration did not differ from that of the " control " livers, and elevated serum levels might therefore occur in these patients. The tumour itself is probably not the source of the serum zinc as the levels are low. Similar values have been reported in hepatic metastases (Olson *et al.*, 1958) which are not associated with raised serum levels (Vikbladh, 1951; Wolff, 1956; Davies, Musa and Dormandy, 1968).

We are indebted to the staff of the National Institute for Research into Occupational Diseases for their co-operation, and to the photographic unit of the Department of Medicine for Fig. 1.

REFERENCES

ADDINK, N. W. H. & FRANK, L. J. P. (1959) Remarks apropos of Analysis of Trace Elements in Human Tissues. *Cancer N.Y.*, **12**, 544.

BECKER, B. J. P. & CHATGIDAKIS, C. B. (1961) Primary Carcinoma of the Liver in Johannesburg. *Acta Un. int. Cancr.*, **17**, 650.

BERMAN, C. (1951) *Primary Carcinoma of the Liver.* London: H. K. Lewis.

BOYETT, J. D. & SULLIVAN, J. F. (1970) Zinc and Collagen Content of Cirrhotic Liver. *Am. J. dig. Dis.*, **15**, 797.

BUTT, E. M. & HIGGINSON, J. (1957) Trace Element Pattern in Liver Disease and Liver Carcinoma. *Acta Un. int. Cancr.*, **13**, 599.

CHAHOVITCH, X. (1955) Action of Zinc on Growth of Experimental Tumours Incited by Carcinogens. *Glasn srp. Akad. Nauka*, **215**, 143.

CHARLTON, R. W., BOTHWELL, T. H. & SEFTEL, H. C. (1973) Dietary Iron Overload. In *Clinics in Haematology.* London: W. B. Saunders. p. 383.

DAVIES, I. J. T. (1972) *The Clinical Significance of the Essential Biological Metals.* London: W. Heinemann. p. 16.

DAVIES, I. J. T., MUSA, M. & DORMANDY, T. L. (1968) Measurements of Plasma Zinc. I. In Health and Disease. *J. clin. Path.*, **21**, 359.

DE WYS, W., PORIES, W. J., RICHTER, M. C. & STRAIN, W. H. (1970) Inhibition of Walker 256 Carcinosarcoma Growth of Dietary Zinc Deficiency. *Proc. Soc. exp. Biol. Med.*, **135**, 17.

GEDDES, E. W. & FALKSON, G. (1970) Malignant Hepatoma in the Bantu. *Cancer, N.Y.*, **25**, 1271.
HIRAYAMA, T. (1962) Quoted in *S. Afr. Cancer Bull.*, **6**, 114.
KOCH, H. J., SMITH, E. R., SHIMP, N. F. & CONNOR, J. (1956) Analysis of Trace Elements in Human Tissues. I. Normal Tissues. *Cancer, N.Y.*, **9**, 499.
LIN, T. Y. (1970) Primary Cancer of the Liver. Quadrennial Review. *Scand. J. Gastroenterol.*, **5**, Suppl. 6, 223.
MACDONALD, R. A. (1957) Primary Carcinoma of the Liver: A Clinico-pathologic Study of 108 cases. *Archs intern. Med.*, **99**, 266.
MCBEAN, L. D., DOVE, J. T., HALSTED, J. A. & SMITH, J. C. (1972) Zinc Concentration in Human Tissues. *Am. J. clin. Nut.*, **25**, 672.
MCGLASHAN, N. D. (1967) Zinc and Oesophageal Cancer. *Lancet*, i, 578.
OLSON, K. B., HEGGEN, G. E. & EDWARDS, C. F. (1958) Analysis of 5 Trace Elements in the Liver of Patients Dying of Cancer and Non-cancerous Disease. *Cancer, N.Y.*, **11**, 554.
PORIES, W. J., HENZEL, J. H., ROB, C. G. & STRAIN, W. H. (1967) Acceleration of Healing with Zinc Sulphate. *Ann. Surg.*, **165**, 432.
POSWILO, D. E. & COHEN, B. (1971) Inhibition of Carcinogenesis by Dietary Zinc. *Nature, Lond.*, **1231**, 447.
SAGEBIEL, R. W., MCFARLAND, R. B. & TAFT, E. B. (1963) Primary Carcinoma of the Liver and Cirrhosis. *Am. J. clin. Path.*, **40**, 516.
STOCKS, P. & DAVIES, R. I. (1964) Zinc and Copper Content of Soils Associated with the Incidence of Cancer of the Stomach and Other Organs. *Br. J. Cancer*, **18**, 14.
VALLEE, B. L., WACKER, W. E. C., BARTHOLOMAY, A. F. & HOCH, F. L. (1957) Zinc Metabolism in Hepatic Dysfunction. II. Correlation of Metabolic Patterns with Biochemical Findings. *New Engl. J. Med.*, **257**, 1055.
VIKBLADH, I. (1951) Studies on Zinc in Blood. *Scand. J. clin. Lab. Invest.*, **3**, Suppl. 2.
WOLFF, H. P. (1956) Untersuchingen zur pathophysiologia des zinkstoffwechsels. *Klin. Wschr.*, **34**, 409.
WRIGHT, E. B. & DORMANDY, T. L. (1973) Liver Zinc in Carcinoma. *Nature, Lond.*, **237**, 166.
YING, Y. Y., MA, C. C., HSU, Y. T., LEI, H. A., LIANG, S. F., LIU, C. H. & KU, C. Y. (1963) Primary Carcinoma of the Liver with Special Reference to Histogenesis and its Relationship to Liver Cirrhosis. *Chin. med. J.*, **82**, 279.